# Online versus in‐person surgical near‐peer teaching in undergraduate medical education during the COVID‐19 pandemic: A mixed‐methods study

**DOI:** 10.1002/hsr2.1889

**Published:** 2024-02-13

**Authors:** Priyanka Iyer, Valerie Mok, Arjan Singh Sehmbi, Nicos Kessaris, Rhana Zakri, Prokar Dasgupta, Pankaj Chandak

**Affiliations:** ^1^ Faculty of Life Sciences and Medicine King's College London London UK; ^2^ Faculty of Medicine University of British Columbia Vancouver British Columbia Canada; ^3^ The Royal London Hospital Barts Health NHS Trust London UK; ^4^ Department of Inflammation Biology, School of Immunology and Microbial Sciences, King's College London Centre for Nephrology, Urology and Transplantation London UK; ^5^ Department and Stem Cell and Regenerative Medicine, Centre for Developmental Biology & Cancer University College London and Great Ormond Street Institute of Child Health London UK; ^6^ MRC Centre for Transplantation, Guy's Hospital King's College London London UK

**Keywords:** clinical education, computers, curriculum development/evaluation, simulation, new technology

## Abstract

**Background and Aims:**

The coronavirus disease 2019 (COVID‐19) pandemic stimulated a paradigm shift in medical and surgical education from in‐person teaching to online teaching. It is unclear whether an in‐person or online approach to surgical teaching for medical students is superior. We aim to compare the outcomes of in‐person versus online surgical teaching in generating interest in and improving knowledge of surgery in medical students. We also aim the quantify the impact of a peer‐run surgical teaching course.

**Methods:**

A six‐session course was developed by medical students and covered various introductory surgical topics. The first iteration was offered online to 70 UK medical students in March 2021, and the second iteration was in‐person for 20 students in November 2021. Objective and subjective knowledge was assessed through questionnaires before and after each session, and also for the entire course. Data were analyzed from this mixed‐methods study to compare the impact of online versus in‐person teaching on surgical knowledge and engagement.

**Results:**

Students in both iterations showed significant improvement of 33%–282% across the six sessions in knowledge and confidence after completing the course (*p* < 0.001). There was no significant difference in the level of objective knowledge, enjoyment, or organization of the course between online and in‐person groups, although the in‐person course was rated as more engaging (mean Likert score 9.1 vs. 9.7, *p* = 0.033).

**Discussion:**

Similar objective and subjective surgical teaching outcomes were achieved in both iterations, including in “hands‐on” topics such as suturing, gowning, and gloving. Students who completed the online course did not have any lower knowledge or confidence in their surgical skills; however, the in‐person course was reported to be more engaging. Surgical teaching online and in‐person may be similarly effective and can be delivered according to what is most convenient for the circumstances, such as in COVID‐19.

## 1 INTRODUCTION

The onset of the coronavirus disease 2019 (COVID‐19) pandemic resulted in a paradigm shift from in‐person to online teaching,^1^, ^2^ triggering academic institutions worldwide to accelerate the development of online learning platforms.^3^ This resulted in two main formats: asynchronous classes, which are recorded lectures or worksheets, and synchronous classes, which are live seminars through video conferencing and virtual classrooms.^3^ Some lectures, tutorials, ward rounds, and consultations became virtual, and both students and teachers had to adapt accordingly often with little preparation time.

Surgical training has historically placed an emphasis on practical skills and experience. The old adage “See one, do one, teach one” traditionally used in surgical training is still somewhat found in modern training.^4^ Although in recent years there has been greater supervisor involvement, the basic principle of repeatedly practising skills *in person*, whether in a simulated or clinical environment, remains essential.^5^ Studies have shown that learning is more efficient when one is physically or mentally involved in the process^6^ and that repeated practice of surgical skills on a model is shown to improve patient safety and trainee confidence.^4^, ^7^ Near‐peer teaching also has proven benefits in developing student confidence and skills in basic surgical education and is commonly seen in medical schools.^8^

Generally, surgical exposure begins in medical school with surgical rotations, after which more advanced surgical training can be undertaken in postgraduate training. In the United Kingdom, this begins at a basic level during the first two “Foundation Training” years and carries on at an advanced level through core and high specialist training.^9^

Numerous studies found that the pandemic had an overall negative impact on medical student education experiences.^10^, ^11^, ^12^ Students commonly report that their experience with surgery and the doctors' engagement andwillingness to teach is what piques a student's initial and continuing interest in a specialty. Given that early‐year medical students have limited placement exposure, each opportunity in a clinic or operating theater becomes a vital opportunity for the surgeons to foster a mentor relationship which creates a lasting impression of the specialty on the student.^13^

### 1.1 Aims and hypothesis

It is unclear whether an in‐person or online approach to surgical teaching for medical students is superior. We aim to compare the outcomes of in‐person versus online surgical teaching in generating interest in and improving knowledge of surgery in medical students. We also aim to quantify the impact of a peer‐run surgical teaching course.

## 2 METHODS

This study follows a mixed‐methods design based on questionnaire responses from participants from a peer‐taught surgical course that included multiple‐choice questions to assess objective knowledge and free‐text response questions to assess engagement.

Introduction to Surgery for Students (ISS) is a peer‐taught course, developed to bolster surgical education in the first and second years of medical school. The course aimed to introduce surgical topics to increase exposure to surgery, educate students about different specialties, and highlight what is needed practically to pursue a surgical career.

The course consisted of six weekly sessions during the evening, each lasting 1.5 h. We delivered two iterations of ISS, held online in March 2021 and held in‐person in November 2021. The session topics were:

1. Course introduction and surgical sieve
2. Guide to theater and perioperative care
3. Basic suturing skills workshop
4. Acute abdomen surgical emergencies and complications
5. Core Surgical Training (CST) portfolio
6. Surgical specialties overview

Instructors were upper‐year medical students (third year and above) who were already involved in the surgical society committee, as well as surgical trainees and consultants from hospitals across the United Kingdom. Didactic topics were taught lecture‐style with group discussion where relevant, such as when describing a patient presentation. The suturing skills workshop and the gowning, gloving, and scrubbing up sections of the “guide to theatre” session were delivered practically in a clinically simulated environment with surgical equipment in the in‐person iteration, in contrast to didactic delivery in the virtual iteration.

Recruitment of participants was done online including through email lists and social media platforms. Participants completed an application form (Supporting Information S1: Appendix Section 1), answering free response texts about their motivation and interest in surgery, and commitment to attending ISS sessions. Participants were selected based on teaching capacity, what they wanted to gain from ISS, and motivation to explore surgery as an option. In March 2021, all sessions were held online using the Zoom video conferencing platform^14^ with unlimited teaching capacity—as such, the course was open to first‐ and second‐year medical students all over the United Kingdom and 70 students were enrolled in the course, where everyone who applied was accepted onto the course. In November 2021, all sessions were held in‐person on a London University campus, but only 20 first‐ and second‐year medical students could be selected due to room social distancing restrictions and on‐campus access. These 20 students were selected by 2 reviewers who independently scored each anonymized candidate response out of 15 (with five marks for the three long answer questions on interest in the course, commitment to surgery, future career plans), and the 20 participants with the highest mean scores were selected.

All feedback from the students and assessment of their knowledge was collected using pseudoanonymized online questionnaires via online forms. In total, students were required to fill in 14 questionnaires, consisting of a pre‐ and postcourse questionnaire, and six sets of pre‐ and postsession questionnaires for each course. Students were required to complete both overall course questionnaires and questionnaires for at least four out of six sessions to receive a certificate.

The same questions were used in both the pre‐ and postsession questionnaires to fairly evaluate the students' progress. These self‐assessments were graded along 5‐ or 10‐point Likert scales, free‐text, or multiple‐choice questions. The questionnaires to assess the students for both the in‐person and online iterations were the same.

In the precourse questionnaire filled in before starting ISS, students rated their confidence in the topics covered in the course and answered questions to objectively assess their understanding of the same topics. The students were then re‐evaluated with the same questions in the postcourse questionnaire. Examples of a questionnaire can be found in Supporting Information S1: Appendix Section 2.

Informed consent to participate in the study was obtained from all students.

### 2.1 Statistics

Qualitative and quantitative data were collected using Google Forms. All statistical analyses were performed using IBM SPSS Statistics (version 28) with results reported as means of Likert scores and *Z*‐scores. The Mann–Whitney *U*‐test was used due to unequal group sizes. A *p* value of less than 0.05 was considered statistically significant.

## 3 RESULTS

There were 70 participants in the online iteration and 20 in the in‐person iteration at the start of the courses, and 56 and 19 participants, respectively, completed the courses (14 and one participants were lost to follow‐up, respectively). In the online iteration, 64.8% of the students were first‐year medical students versus 30% in the in‐person iteration. On a Likert scale of 1–10, with 1 = *not interested* and 10 = *very interested*, the mean score in the online iteration was 9.1, and 8.2 in the in‐person iteration. In total, 78.4% of students online and 100% of students in‐person felt that they had unsatisfactory exposure to surgery, with most reporting they had minimal exposure.

**Figure 1:**
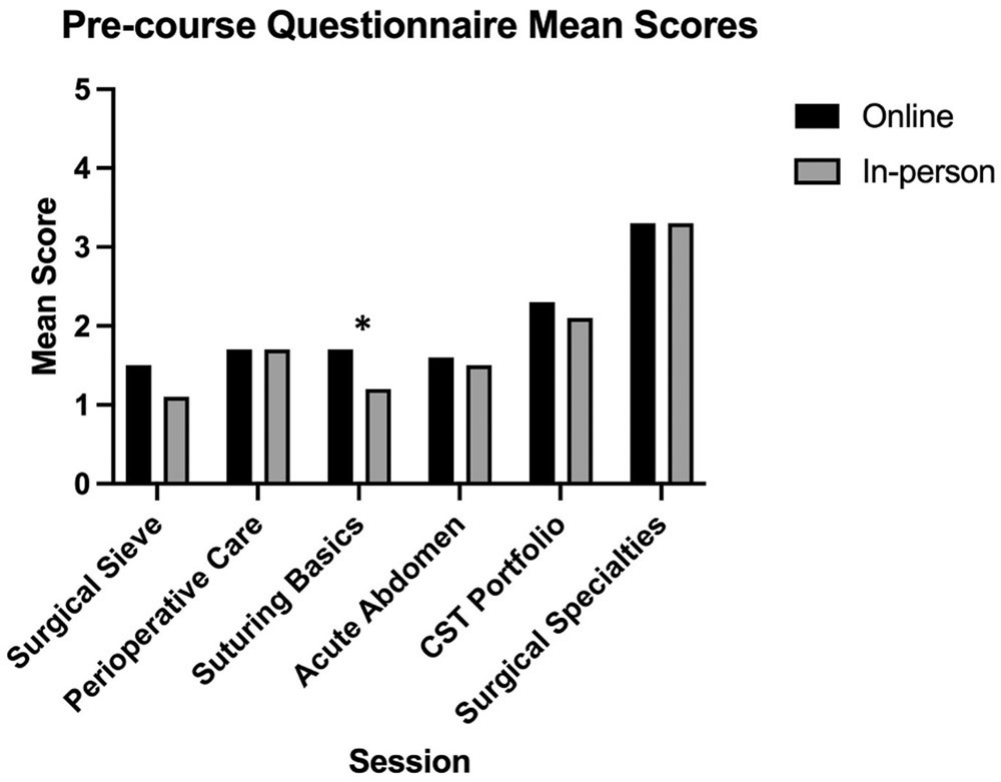
A comparison of precourse questionnaires for both iterations indicating students' baseline levels of knowledge. Questionnaire scores were reported as mean Likert scores out of 5. Asterisk indicates that online students had a greater mean score than in‐person students (*p* = 0.007). CST, Core Surgical Training.

In our study, students in both iterations showed a significant improvement (*p* < 0.001) in both knowledge and confidence regarding all the sessions' topics based on pre‐ and postcourse questionnaires (Table 1 and Figure 1). Across all sessions, in‐person and online, there was a 33% to 282% improvement in objective knowledge and confidence before and after the course for each session. They also showed a significant improvement (*p* ≤ 0.01) in baseline knowledge from before to after each session in both iterations.

**Table 1:** Mean likert scores showing improvement in knowledge and confidence in all sessions, based on questionnaires.

	Precourse questionnaire	Postcourse questionnaire	Percent change	*Z*‐score	*p* Value
Online sessions	(*n* = 70)	(*n* = 56)			
Surgical sieve	1.5	4.1	173	9.36	<0.001
Perioperative care	1.7	4.0	135	7.88	<0.001
Suturing basics	1.7	3.7	118	7.96	<0.001
Acute abdomen	1.6	4.0	150	9.09	<0.001
CST portfolio	2.3	4.2	83	7.81	<0.001
Surgical specialties	3.3	4.4	33	6.25	<0.001
In‐person sessions	(*n* = 20)	(*n* = 19)			
Surgical sieve	1.1	4.2	282	5.81	<0.001
Perioperative care	1.7	3.9	129	5.34	<0.001
Suturing basics	1.2	4.0	233	5.70	<0.001
Acute abdomen	1.5	3.9	160	5.36	<0.001
CST portfolio	2.1	3.9	86	4.47	<0.001
Surgical specialties	3.3	4.4	33	4.21	<0.001

*Note*: Questionnaire scores were reported as mean Likert scores out of 5.

Abbreviation: CST, Core Surgical Training.

On comparing the precourse questionnaires, there was no statistically significant difference in the baseline knowledge between both the online and in‐person groups for all sessions except suturing, where the online group had a greater mean score than the in‐person group (*p* = 0.007) as seen in Figure 1.

**Figure 2:**
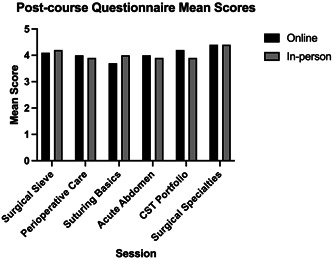
A comparison of postcourse questionnaires for both iterations indicated that the students had similar levels of knowledge of course completion. Questionnaire scores were reported as mean Likert scores out of 5.

The study also showed that there was no significant difference in the mean level of knowledge between groups at course completion (Figure 2).

**Figure 3:**
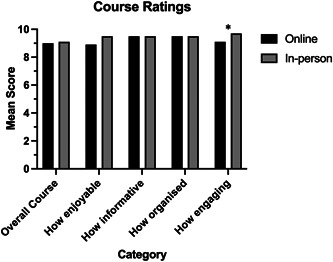
A comparison of overall ratings of the online and in‐person courses. Asterisk indicates that in‐person students reported a greater mean score than online students (*p* = 0.03).

There were also no significant differences between the ratings given to each iteration regarding the course overall and how enjoyable, informative, and organized it was. However, the in‐person iteration was significantly more engaging (*p* = 0.033) than the online iteration (Figure 3).

On the free‐text responses regarding the advantages of online learning, 91.9% of participants felt that accessibility and flexibility were key advantages and 29.0% felt that ease of participation, such as being able to ask and answer questions via chat, was an advantage. A total of 9.7% of participants felt that online courses are more efficient (could cover more content at a faster rate) and could have a greater range of speakers from various locations or time zones.

Free‐text responses on the advantages of in‐person learning showed that 100% of participants found it more engaging or enjoyable; this is supported by the in‐person cohort rating our in‐person course as more engaging. In total, 73.4% of participants felt comfortable participating in discussions; 42.1% mentioned practical hands‐on opportunities; 31.6% felt that they could get instant feedback on their work or questions; 21.1% felt they retained information better; and 15.8% mentioned more networking opportunities.

## 4 DISCUSSION

In this study, students' knowledge of surgery improved through the course, with significant improvement in the participants' objective knowledge for both online and in‐person iterations. Additionally, students' subjective confidence in their knowledge also increased. Although students found the in‐person course more engaging (mean score 9.1 vs. 9.7, *p* = 0.033), the outcomes between the virtual and in‐person iterations were not significantly different, suggesting that the style of teaching engagement does not substantially impact the knowledge gained.

Conducting courses online can have various advantages such as schedule flexibility, cost‐effectiveness, and the opportunity to learn from peers, institutions, and professors around the world.^15^, ^16^ The main advantage reported by the participants was the ease of accessibility, and we accommodated students across the United Kingdom and internationally. This means surgical education can be made more accessible to less economically developed areas that may not have access to resources like suturing equipment or models. Many students also found contributing via the Zoom chat function^14^ less intimidating and disruptive to the tutorial and to fellow students.

In‐person learning also has several advantages, such as being more engaging and interactive. Our online participants found networking with other students or speakers difficult, as most students kept their webcam and microphone off. However, in the in‐person iteration, students were able to engage in discussion with peers and speakers more easily and readily. Hosting the course in‐person allowed students to perform the relevant practical skills whilst our team of supervisors corrected their technique and provided assistance in real‐time. During the pandemic, a lack of hands‐on practice for medical students and junior doctors led to a reduction in confidence and competence.^15^, ^17^ In our online course, many students reported that they would have preferred to do the practice suturing in person to familiarize themselves with surgical instruments or appreciate the relationship between three‐dimensional anatomical structures.

Although students in‐person found the course more engaging, both iterations were rated as similarly enjoyable, informative, and well‐organized, and students had similar levels of quantitative knowledge at the end of each course. A hybrid model would be most effective in improving student outcomes in terms of both engagement and knowledge as it allows for flexibility in selecting activities suitable to the modality, whether in‐person or online.^18^ For example, practical sessions such as suturing, gowning, and gloving that require experiential hands‐on learning can be conducted in‐person. Knowledge‐based sessions on the surgical sieve, acute abdominal emergencies, CST portfolio, and surgical specialties have a didactic component but still require discussion and could have external speakers—these sessions could be effectively facilitated online. Moreover, technology can be utilized to record sessions and collect feedback via online forms, distribute handouts, and email session reminders. Simulation can also be used to allow students to learn how to use surgical equipment without endangering patients and simultaneously minimizing COVID transmission risk.^19^ Future courses could incorporate a virtual operating room to complete teaching on surgical scrubbing without having to enter a live operating theatre,^20^ or a virtual reality simulation of exploring the anatomy of an acute abdominal presentation. This would help students bring their learning from the classroom into the context of clinical practice, but the initial cost to acquire virtual reality headsets may be prohibitive. Laparoscopic surgery simulation could also complement in‐person suturing sessions,^21^ providing students with an opportunity they would not otherwise be able to attain.

### 4.1 Study strengths and limitations

A strength of our study was that the content, structure, and teaching styles of both courses were similar, allowing for appropriate comparison between cohorts, despite the uneven cohort size. Questionnaires were also rigorously applied before and after each session and the entire course, strengthening the causal relationship of the change in objective and subjective scores to the sessions themselves.

A limitation of our study was the selection bias in recruiting participants, as well as the difference in recruitment approaches for both cohorts. The online cohort was much larger, accepting everyone who applied regardless of the quality of application. Whereas the in‐person cohort was recruited using a subjective method to score applicants, selecting only the 20 best applicants, due to inevitable COVID social distancing requirements. However, the baseline scores of knowledge in both cohorts were mostly similar. There was also a greater proportion of first‐year students in the online course and more second‐year students in‐person. We required students to attend four of six sessions to complete the course to mitigate nonattendance, but more students were lost to follow‐up or did not complete the course in the online iteration; this may be due to the 20‐person limitation for the in‐person course. The order of tutorials varied minimally between groups.

## 5 CONCLUSION

In conclusion, the pandemic marked the dawn of online learning in medical schools, including in surgical education. We found that both online and in‐person surgical teaching were similar in terms of gaining subjective and objective surgical knowledge, and in the participants' enjoyment of the course. In our course, there was no significant difference in the amount of knowledge gained in both versions of the course. We also established that although the students found the in‐person course more enjoyable and engaging, they were both similarly informative and well‐organized. Depending on the individual circumstances, an online or in‐person can be used with similar expected benefits to students. Given our study population consisted of medical students from across the country, we believe our study's results may be generalizable to other student‐led near‐peer surgical courses in a variety of online and in‐person settings. Further studies are warranted in other countries and with larger sample sizes with different student or trainee populations to confirm our findings. For future iterations of surgical courses, we propose that a hybrid model that combines elements of both approaches be adopted to provide an ideal method for surgical teaching.

## AUTHOR CONTRIBUTIONS

**Priyanka Iyer**: Conceptualization; data curation; methodology; writing—original draft; writing—review and editing. **Valerie Mok**: Conceptualization; data curation; methodology; writing—original draft; writing—review and editing. **Arjan Singh Sehmbi**: Methodology; supervision; writing—review and editing. **Nicos Kessaris**: Writing—review and editing. **Rhana Zakri**: Writing—review and editing. **Prokar Dasgupta**: Writing—review and editing. **Pankaj Chandak**: Conceptualization; supervision; writing—review and editing.

## CONFLICT OF INTEREST STATEMENT

Prokar Dasgupta is affiliated with Proximie and MysteryVibe. The remaining authors declare no conflict of interest.

## TRANSPARENCY STATEMENT

The lead author Priyanka Iyer affirms that this manuscript is an honest, accurate, and transparent account of the study being reported; that no important aspects of the study have been omitted; and that any discrepancies from the study as planned (and, if relevant, registered) have been explained.

## Supporting information

Supporting information.

## Data Availability

The data that support the findings of this study are available from the corresponding author upon reasonable request.
