# Supplementary material for: Online versus in‐person surgical near‐peer teaching in undergraduate medical education during the COVID‐19 pandemic: A mixed‐methods study
Source: Health Sci Rep. 2024 Feb 13;7(2):e1889. doi: 10.1002/hsr2.1889 (PMC10864811; doi:10.1002/hsr2.1889)
Supplement: Supplementary file 1 — Supporting information. [file HSR2-7-e1889-s001.docx]

Appendices

1. Application form (in-person iteration, Nov 2022)
2. Post-course questionnaire (same for both iterations)

1. Application form (in-person iteration, Nov 2022)

The course outline is as follows:

Week 1 - Course Introduction and Surgical Sieve

Week 2 - Guide to Theatre and Perioperative Care

Week 3 - Basic Suturing Skills Workshop

Week 4 - Acute Abdomen Surgical Emergencies and Complications

Week 5 - Core Surgical Training (CST) Portfolio

Week 6 - Surgical Specialties Overview

1. What is your full name?
2. What is your student ID number?
3. What is your email?
4. What MBBS year are you in?
5. Why are you interested in attending this course? What would you like to gain from this course? (Max. 150 words)
6. What steps have you taken, if any, to show your commitment to surgery? (Max. 150 words)
7. Are you considering surgery as a career? If yes, what specialty? (Max. 150 words)
8. Are you able to commit to attending at least 5 out of 6 sessions and filling out the relevant feedback forms?

Your responses in this section will NOT affect your chances of being accepted onto the course, so please answer truthfully. This will help us in our research to better understand the gaps in the KCL MBBS surgical teaching and whether courses like this will help reduce that gap.

1. How interested are you in pursuing surgery? (1-not interested, 10-extremely interested). Mark only one oval.
2. How much exposure have you had to surgery so far? Mark only one oval.

- None
- Minimal
- Some but not satisfactory
- Satisfactory
- More than enough

1. How satisfied are you with the level of surgical teaching in the current MBBS Curriculum? Mark only one oval. (1-Very dissatisfied, 10-very satisfied)
2. What is your preferred learning style for surgical teaching? (Choose up to 3)

- Online lectures
- In-person lectures
- Online tutorials
- In-person tutorials
- Videos
- Practical workshops
- Clinical placements

1. Do you prefer in-person or online teaching? Please provide three reasons why.
2. How confident are you in your knowledge of how to build a strong portfolio for surgical training entry? (1-not confident, 10-very confident). Mark only one oval.

2. Post-course Questionnaire

1. Full Name (your responses will remain anonymous; this is to prevent duplicates)
2. What year are you in?
3. Rate the course: Mark only one oval. (1-unsatisfactory , 10-excellent)
4. How enjoyable was the course? Mark only one oval. (1-not enjoyable at all, 10-very enjoyable)
5. How informative was the course? Mark only one oval. (1-not informative at all, 10-very informative)
6. How well-organised was the course? Mark only one oval. (1-not organised at all, 10-very organised)
7. How engaging were the teachers? (Valerie and Priyanka) Mark only one oval. (1-not engaging at all, 10-very engaging)
8. The content was appropriate for your level. Mark only one oval. (Strongly agree, agree, neutral, agree, strongly agree)
9. The content was delivered at an appropriate pace. Mark only one oval. (Strongly agree, agree, neutral, agree, strongly agree)
10. What was good about the course? (short answer question)
11. Were your expectations of the course met? (Yes, no, not sure)
12. Rate your confidence in forming a differential diagnosis using a surgical sieve: Mark only one oval. (1-not confident, 5-very confident)
13. Rate your confidence in scrubbing up and knowledge on perioperative care (e.g. anaesthesia and WHO surgical safety checklist): Mark only one oval. (1-not confident, 5-very confident)
14. Rate your confidence in building your Core Surgical Training (CST) portfolio in medical school: Mark only one oval. (1-not confident, 5-very confident)
15. Rate your confidence in basic suturing skills (e.g. types of suture materials, ties, knots, indications etc.): Mark only one oval. (1-not confident, 5-very confident)
16. Rate your knowledge of acute surgical abdomen presentations and their complications: Mark only one oval. (1-not confident, 5-very confident)
17. Rate your awareness of different surgical specialty careers: Mark only one oval. (1-not confident, 5-very confident)
18. Do you think this course was limited by being in-person? If so, how? (short answer question)
19. What are up to 3 advantages of in-person learning? (short answer question)
20. What are up to 3 disadvantages of in-person learning? (short answer question)
21. How can the course be improved for future iterations (both if online or in-person)? (short answer question)
22. Do you prefer online or in-person classes, and why? (short answer question)
23. Any other comments/ feedback? (short answer question)
